# Lethal Transaldolase Deficiency Mimicking Gestational Alloimmune Liver Disease: Prenatal Sonography and Identification of a Novel *TALDO1* Variant

**DOI:** 10.1055/a-2946-5351

**Published:** 2026-09-18

**Authors:** Tiara M. Adeniji, Jason Carmichael, Sanmaan Basraon, James Hole, Bryce A. Mendelsohn, Beni A. Adeniji

**Affiliations:** 1 Department of Health Science Studies 667952 Robbins College of Health and Human Sciences, Baylor UniversityWacoTexasUnited States; 2 71437 Maternal Fetal Center, Valley Children’s HealthcareMaderaCaliforniaUnited States; 3 Department of Genetics 6470 Kaiser Permanente Health CareOaklandCaliforniaUnited States

**Keywords:** transaldolase deficiency, *TALDO1* variant, prenatal diagnosis, neonatal hemochromatosis, gestational alloimmune liver disease, fetal growth restriction, echogenic bowel, exome sequencing

## Abstract

**Objective** The objective of this study is to describe the prenatal sonographic and neonatal features of two siblings with lethal transaldolase deficiency and highlight its phenotypic overlap with gestational alloimmune liver disease (GALD).

**Study Design** This is a retrospective review of consecutive pregnancies in a single family, analyzing sequential prenatal ultrasound surveillance, maternal therapeutic interventions, neonatal clinical course, and postmortem trio exome sequencing.

**Results** Both siblings exhibited severe early-onset fetal growth restriction, echogenic bowel, neonatal hemochromatosis, and hypertrophic cardiomyopathy. Because the clinical presentation during the first pregnancy closely mimicked GALD, the second pregnancy was treated empirically with intravenous immunoglobulin (IVIG), which proved entirely ineffective. Subsequent exome sequencing revealed compound heterozygous variants in the *TALDO1* gene, including a novel pathogenic variant (p.Arg239Cys). These findings demonstrate that the hepatic pathology in transaldolase deficiency is a primary metabolic process rather than an antibody-mediated alloimmune process.

**Conclusion** Transaldolase deficiency should be considered in the differential diagnosis for fetuses presenting with unexplained severe growth restriction accompanied by echogenic bowel or hepatic echogenicities or in neonates with fulminant liver failure, particularly when associated with cardiomyopathy. This case highlights the value of performing prenatal exome sequencing prior to initiating targeted therapies like IVIG. Establishing a definitive genetic diagnosis prevents the utilization of futile, resource-intensive treatments, optimizing patient care and prognostic counseling.

## Introduction

Transaldolase deficiency (OMIM 606003) is a rare, autosomal-recessive inborn error of metabolism affecting the pentose phosphate pathway (PPP). It is caused by homozygous or compound heterozygous pathogenic variants in the *TALDO1* gene 1 located on chromosome 11 *p* 15. The PPP functions to generate reducing equivalents in the form of NADPH, ribose-5-phosphate, and pentoses for the glycolytic pathway, 2 which in turn are used for the synthesis of lipids, ATP, GSH, and many other pathways. 3 It is one of the body’s main antioxidant cellular defense systems. Transaldolase is vital in the nonoxidative part of the PPP after transketolase, 4 and the absence or deficiency of transaldolase causes accumulation of products such as ribitol, erythritol, and sedoheptulose, causing variable effects ranging from severe prenatal findings such as hydrops fetalis, oligohydramnios, and in utero lethality to neonatal cirrhosis, liver failure, tubulopathy, dysmorphia, and hematologic defects such as anemia and thrombocytopenia. 5 Prenatal diagnosis is very challenging as the prenatal findings can be nonspecific. 6 Diagnosis of transaldolase deficiency is often not possible until the postnatal period and TALDO deficiency can have significant variability in clinical manifestations and outcomes even within the same family. 6 7 8

In this report, we describe the prenatal sonographic features as well as severe neonatal deterioration in two siblings with neonatal hemochromatosis, initially thought to be gestational alloimmune liver disease (GALD) and hypertrophic cardiomyopathy. Exome sequencing identified a novel variant in the *TALDO1* gene that, to the best of our knowledge, had not previously been described as pathogenic.

## Materials and Methods

### Subjects

#### First Pregnancy

The mother was referred for a small for gestational age fetus. On the fetal anatomy survey at 20 weeks of gestation, fetal echogenic bowel, mild bowel dilation, and mild polyhydramnios (deepest vertical pocket 8.4 cm) were noted. The couple was not consanguineous and had no significant medical or genetic disease history.

At our sonogram at 32 weeks of gestation, the fetus was asymmetrically growth restricted based on abdominal circumference less than the 5th percentile. Overall estimated fetal weight (EFW) of 1712 g was still at the 29th percentile (Hadlock) for 32 weeks of gestation. The amniotic fluid index was normal at 14.5 cm with the largest vertical pocket of 4.63 cm. The bowel was noted to show grade 2 echogenicity. 9 10 The material within the bowel lumen was more echogenic than is usual for this gestational age, and the transverse colon was mildly dilated. The transverse colonic diameter of 1.21 cm was above the 90th percentile for the gestational age. 11 There was also mild placental thickening, measuring 7.7 cm in diameter. At 35 ^6/7^ weeks of gestation, the EFW was 1,996 g (<10th percentile), and the abdominal circumference was 27.65 cm (<3rd percentile). An amniotic fluid index of 4.4 cm and the deepest vertical pocket of 1.9 cm indicated oligohydramnios.

The female infant was delivered at 36 weeks, weighing 1820 g (5th percentile). Reticulocyte count on the first day was elevated at 9%. The platelet count at birth was 33,000/µL 3 ; prothrombin time (PT)/partial thromboplastin time (PTT) was abnormal (PT/international normalized ratio 48.5 s/5.2) and the neonate had hypofibrinogenemia at 147 mg/dL (200–400 mg/dL). There was no metabolic acidosis. The newborn received multiple transfusions of fresh-frozen plasma, platelets, and packed red blood cells for disseminated intravascular coagulation.

Physical exam and imaging showed a decrease in subcutaneous fat consistent with intrauterine growth restriction, hypotonia, butterfly vertebra at T5, a small umbilical hernia, female external genitalia with hypoplastic labia, external rotation at the hips, boggy fontanelles, hirsute forehead, short palpebral fissures, telecanthus, and a broad nasal bridge. Blood was sent for karyotyping and chromosomal microarray (CMA) analysis. These demonstrated Trisomy X and a small 373-kb duplication at chromosome 16p13.3 of uncertain significance.

Evaluation for neonatal alloimmune thrombocytopenia was negative, and maternal antiplatelet antibodies were negative. Serum ferritin in the neonate was 920 ng/mL (normal: 25–200 ng/mL).

The infant developed conjugated hyperbilirubinemia and by day 20: total bilirubin was 11.6 mg/dL with a conjugated fraction of 6.5 mg/dL (110.5 μmol/L). Despite phenobarbital and phototherapy, the bilirubin level on day 32 was 25.9 mg/dL with a conjugated fraction of 21 mg/dL.

On day 32, ultrasound showed splenomegaly and ascites. An echocardiogram on day 33 showed hypertrophic cardiomyopathy with no evidence of outflow tract obstruction, normal segmental intracardiac anatomy, no evidence of patent ductus arteriosus, no pericardial or pleural effusion, but ascites was noted. The neurologic exam was notable for profound hypotonia and unresponsiveness to stimuli. No seizures were seen on electroencephalography.

On day 33, the family opted to withdraw care. An autopsy showed a markedly abnormal liver with relatively little normal liver tissue present. The hepatic iron concentration was 6275.9 μg/g (normal: 200–1600). On microscopy, the liver architecture was transformed into micronodules with pseudoacinar structures containing intracanalicular/intraductular cholestasis. A presumptive diagnosis of neonatal hemochromatosis ascribed to GALD was made.

#### Second Pregnancy

During the second pregnancy, with the same partner, maternal testing showed a normal karyotype and did not identify the 16p13.3 duplication that was seen in the first pregnancy; paternal testing could not be performed prior to conception. A plan was made to utilize intravenous immunoglobulin (IVIG) in this pregnancy based on the diagnosis of GALD. 12

**Fig. 1:**
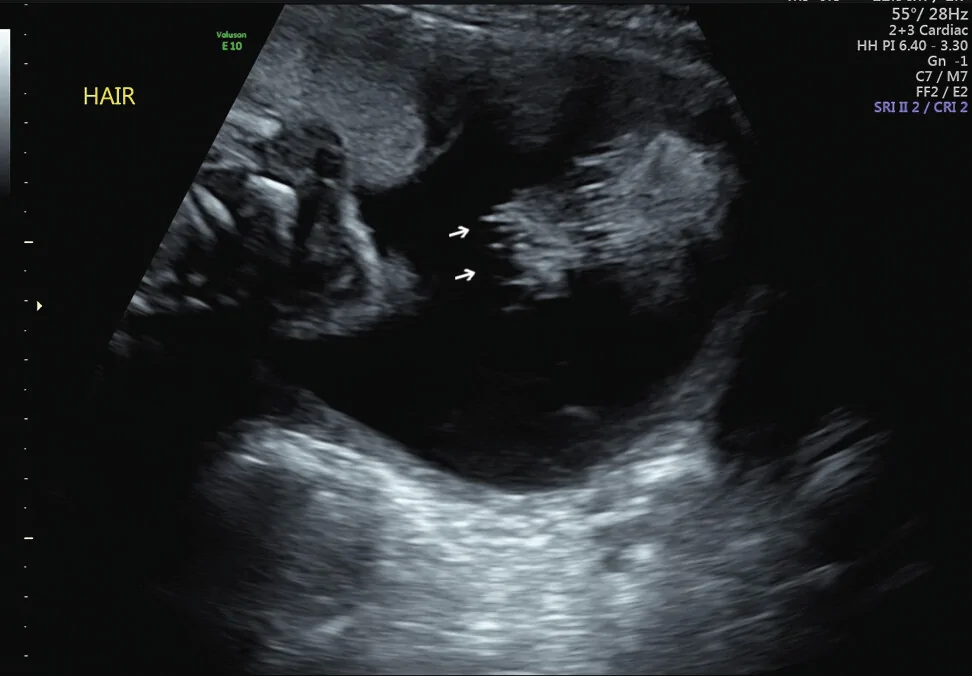
Fetal hypertrichosis (excessive hair growth on fetal scalp) at 27 weeks of gestation. Arrows point to excessive fetal scalp hair.

**Fig. 2:**
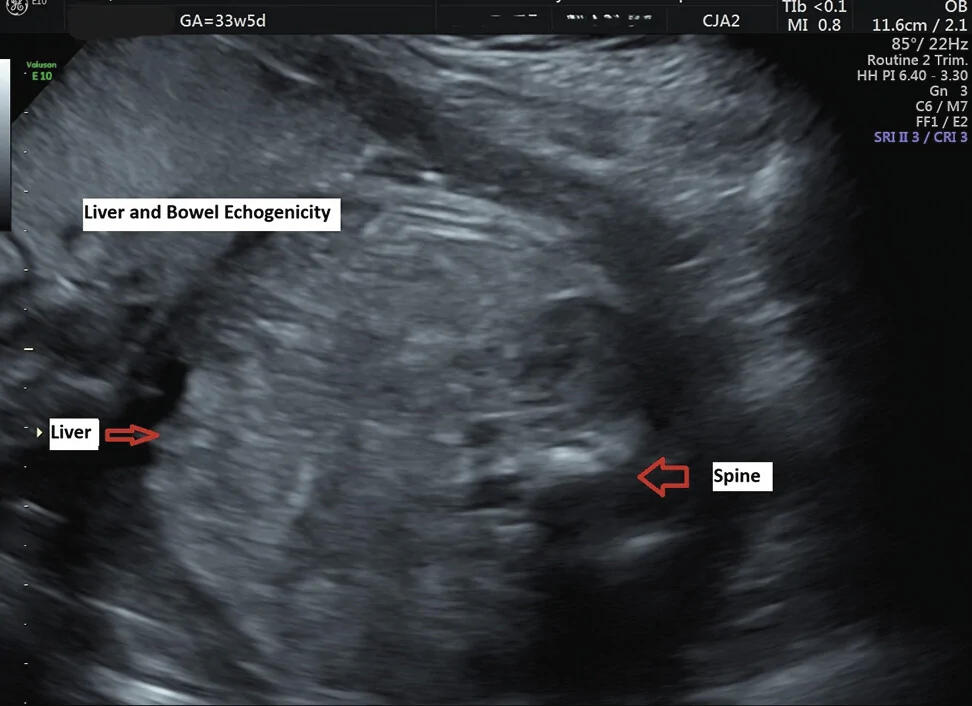
Echogenic bowel and liver. Transverse sonogram through fetal abdomen at 33 weeks of gestation. Increased echogenicity seen in the liver and also bowel as bright as the bone of the fetal spine in several areas. Arrows point to echogenicities in the fetal liver, and the spine is shown as a reference.

**Fig. 3:**
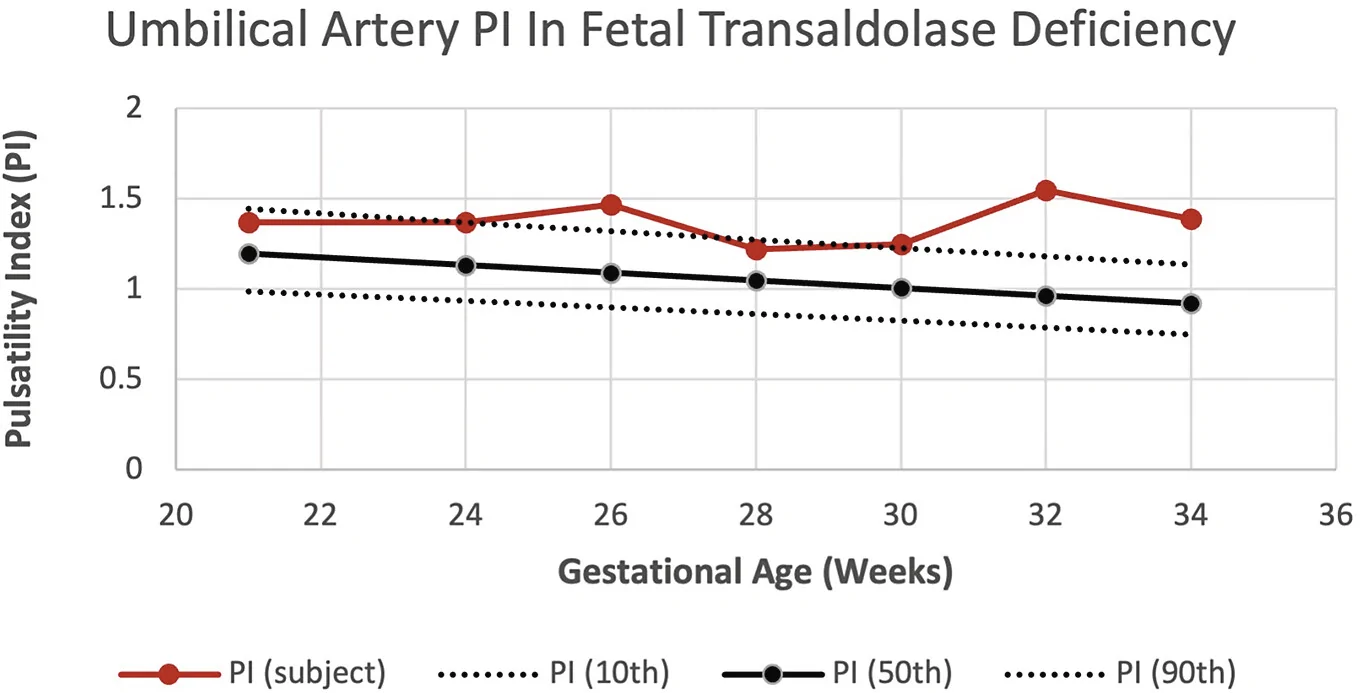
Graph of pulsatility index in subject number 2 (plotted against gestational age normal).

Early sonogram confirmed the gestational age and normal first trimester anatomy. The weekly IVIG therapy was commenced at 14 weeks. At 17 weeks, the fetal size was lagging with biometry at 10 days behind and increased bowel echogenicity was observed. An ultrasound-guided amniocentesis performed at 19 weeks showed a normal male microarray result with no copy number variants and a negative ToRCH polymerase chain reaction for infection. At 28 weeks, there was mild retrognathia, prominent scalp hair (hypertrichosis; Fig. 1 ), increased bowel echogenicity ( Fig. 2 ), an amniotic fluid index of 7.5 cm, and increased umbilical artery (UA) pulsatility index (PI) on UA doppler velocimetries ( Fig. 3 ). At 33 weeks, the placenta was noted to be mildly thickened at 5.87 cm. The UA PI at 33 weeks was now above the 95th percentile for age. 13

The male infant was eventually born at 37 weeks of gestation and was severely growth restricted with a birth weight of 1605 g (less than 1st percentile). Exchange transfusion was conducted due to concern for an alloimmune process. He had hypoglycemia intermittently with poor weight gain and worsening liver enzyme testing. On day 30, his liver profile was grossly abnormal, with evidence of significant cholestasis. ALT 82 (20–60 U/L), alkaline phosphatase 994 (82–383 U/L), total bilirubin is 46.0 mg/dL (0.2–1.3 mg/dL), direct bilirubin 26.5 mg/dL (normal is <0.3 mg/dL). Abdominal sonogram showed stones and sludge in the neonate’s common bile duct and cystic duct, magnetic resonance imaging of the abdomen identified diffuse signals within the liver and a 1.5-cm subcapsular mass. Moderate hemochromatosis was noted with estimation of iron concentration between 6.9 and 8.9 mg/g of liver dry weight.

**Fig. 4:**
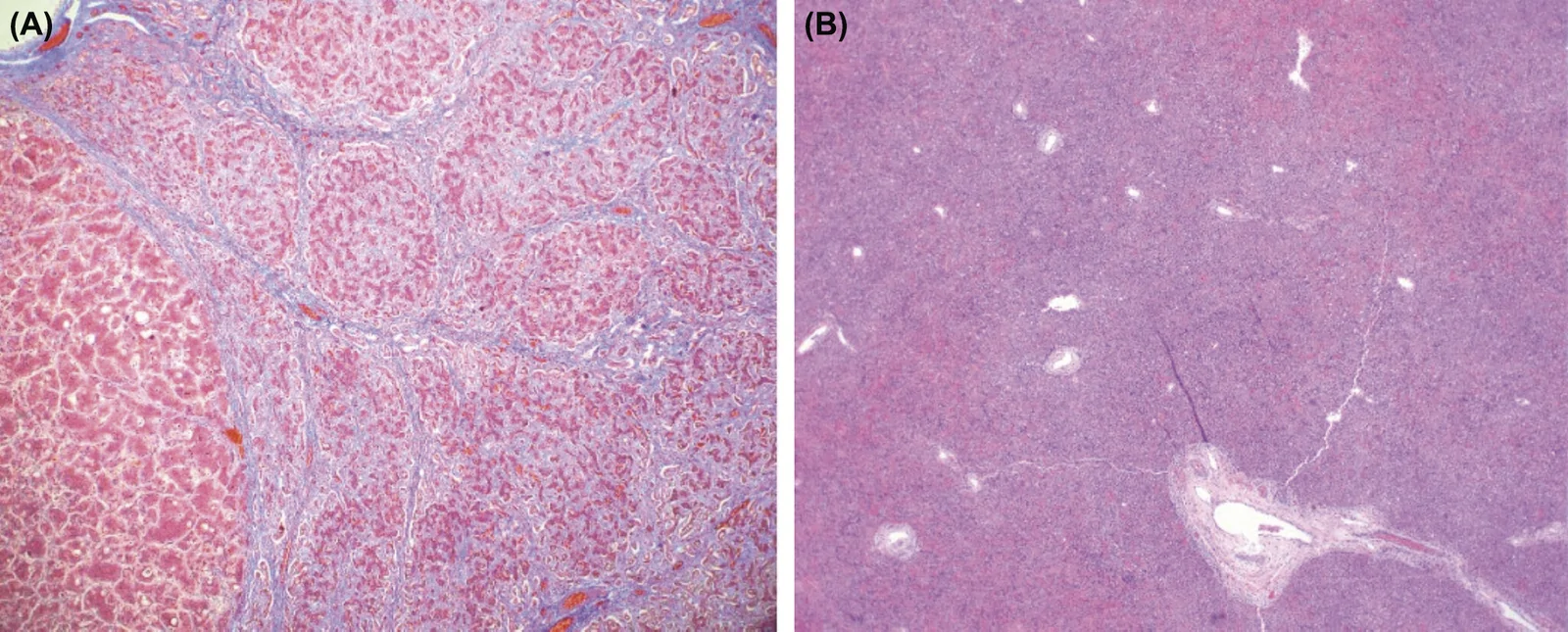
Histopathology slides. ( **A** ) Autopsy slides of liver section for our patient: trichrome, 4×. Low-power view demonstrating prominent nodularity and increased fibrosis (stained in blue) even within the nodules. The large macronodule on the bottom left shows much less fibrosis. ( **B** ) Age-matched normal liver section: liver from a different age-matched autopsy case demonstrating mostly normal histology. Portal area is present and sinuses demonstrate blood and prominent extramedullary hematopoiesis.

A mitochondrial DNA depletion and hepatopathy gene panel was completed with no diagnostic findings. The infant developed worsening coagulopathy and intracranial hemorrhage. At day 43, the parents opted to withdraw care. The autopsy showed neonatal hemochromatosis as well as hypertrophic cardiomyopathy. Fig. 4 shows histopathology slides of the liver for this neonate ( Fig. 4A ) compared with age-matched control ( Fig. 4B ).

After the death of the second affected newborn, trio-based exome sequencing was performed (Fulgent Genetics).

For the deceased sibling from 4 years prior, formalin-fixed paraffin-embedded tissue/slides was utilized for genomic DNA isolation (Fulgent Genomics). DNA was amplified for the target region and sequenced bidirectionally using an ABI 3730XL high-throughput DNA analyzer for Sanger sequencing.

## Results

**Fig. 5:**
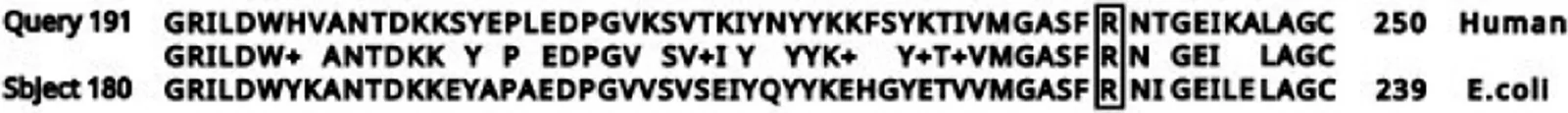
Alignment of human and *Escherichia coli* *TALDO1* .

On the trio-based exome sequencing, two variants in trans were reported in the *TALDO1* gene. Biallelic pathogenic variants in *TALDO1* are associated with transaldolase deficiency (PubMed:19401148; OMIM: 602063). The paternally inherited variant, NM_006755.1:c.574C>T (p.Arg192Cys), has been identified in the homozygous state in multiple patients with transaldolase deficiency and is considered pathogenic. The maternally inherited heterozygous variant, NM_006755.1: c.715C>T (p.Arg239Cys) has not been previously reported as pathogenic. This variant has been observed at a frequency of less than 0.01% (20/246152 alleles) across the Broad gnomAD dataset. 14 The variant is highly suspicious, because it is conserved throughout evolution, including bacteria ( Fig. 5 ), and has been reported to form part of the phosphate-binding site of the enzyme. Polyol analysis could not be performed, because the transaldolase diagnosis was identified after the death of both affected infants.

The mother had a third pregnancy with a different partner without using IVIG therapy, and there were no prenatal growth complications; she gave birth to a healthy term female baby.

## Discussion

We present the prenatal sonographic features of two siblings with courses marked by severe early-onset fetal growth restriction, echogenic bowel, placentomegaly, 15 and late-pregnancy oligohydramnios. Postnatally, both siblings developed severe neonatal hemochromatosis and hypertrophic cardiomyopathy, culminating in lethality. Genetic analysis identified compound heterozygous *TALDO1* variants—including a novel, maternally inherited variant (p.Arg239Cys) located in the phosphate-binding site, which was found in trans with a known pathogenic variant (p.Arg192Cys).

The p.Arg239Cys variant is present in population databases (rs201836196, gnomAD 0.04%) but had not previously been reported in the literature in individuals affected with *TALDO1* -related conditions. ClinVar contains an entry for this variant (Variation ID:2413011). Invitae Diagnostic Test Evidence Modeling of protein sequence (Labcorp Genetics) indicate that this missense variant is expected to disrupt *TALDO1* protein function with a positive predictive value of 80%. Our findings suggest that this variant is pathogenic based on the phenotype, presence in trans with a pathogenic variant, and high evolutionary conservation with protein modeling localizing the affected arginine to the phosphate-binding domain of the protein.

This diagnosis was missed in the first pregnancy, due to a nondiagnostic CMA, leading to a presumed diagnosis of GALD, which dictated management in the subsequent pregnancy. GALD is the leading cause of neonatal acute liver failure and results from maternal IgG-mediated complement activation. As an alloimmune process, it is highly responsive to prenatal IVIG and postnatal exchange transfusion. By contrast, transaldolase deficiency is a chronic, progressive metabolic disease lacking disease-specific therapy.

Valayannopoulos et al. 16 described four patients with transaldolase deficiency and postulated that GALD could be a secondary mechanism of injury in these cases; however, our experience suggests otherwise. In our pregnancy, empiric prenatal treatment with IVIG and postnatal exchange transfusion was entirely ineffective, indicating that the hemochromatosis seen in transaldolase deficiency is likely a primary, nonimmune metabolic process. Because both hereditary and alloimmune disorders carry high recurrence risks in subsequent pregnancies, transaldolase deficiency must be considered as a differential diagnosis along with GALD in a newborn presenting with neonatal hemochromatosis and fulminant liver failure, especially when associated with cardiomyopathy, which is not typically reported with GALD. 17

Standard diagnostic guidelines for severe early-onset fetal growth restriction currently recommend amniocentesis with CMA testing. However, CMA is insufficient to detect single-gene metabolic disorders such as transaldolase deficiency. Recent literature demonstrates that while the incremental yield of prenatal exome sequencing over CMA is 4% in isolated growth restriction, it rises to 30% when accompanied by multisystem anomalies. 18 Although the findings in our cases (echogenic bowel, hepatic echogenicity, elevated UA PIs, placentomegaly and hypertrichosis are not classic structural malformations, they constitute a highly unusual multisystem constellation and should prompt a whole-exome sequencing on prenatal testing.

Our cases also highlight the necessity of using whole-exome sequencing to identify pathogenic single-gene metabolic disorders, especially *TALDO1* variants, before presuming a GALD etiology in a newborn with neonatal hemochromatosis, as these conditions present with a very similar phenotype in the immediate neonatal period. This distinction is critical, as the management, counseling, and prognosis for a subsequent pregnancy will differ fundamentally.

Available therapeutic options for transaldolase deficiency remain mostly supportive. Rodan and Berry 19 demonstrated normalization of serum α-fetoprotein levels in an infant with transaldolase deficiency using *N* -acetylcysteine therapy; however, there is no specific curative therapy available. Ultimately, while prenatal diagnosis of transaldolase deficiency may not avert lethality or reverse the underlying clinical course, it is vital for providing accurate prognostic counseling, guiding complex postnatal management decisions, and identifying potential candidates for early liver transplantation. 20

Limitations of this study include a single family and, because the transaldolase diagnosis was made postmortem, the lack of polyol and other biochemical studies on an affected proband.
